# Supplementary material for: Model Selection Reveals the Butyrate-Producing Gut Bacterium Coprococcus eutactus as Predictor for Language Development in 3-Year-Old Rural Ugandan Children
Source: Front Microbiol. 2021 Jun 2;12:681485. doi: 10.3389/fmicb.2021.681485 (PMC8206532; doi:10.3389/fmicb.2021.681485)

Supplemental file 2

Mixed integer optimization (MO) of the gut microbiota data at the genus level.

Figure 1. Predictors for the 10 best fitting models of 1 up to 4 terms linking language development of children aged 36 months to membership of the intervention or control group, values of 6 developmental parameters of the same children when aged 20-24 months, their microbiota composition at that age, and microbiota composition at 36 months. Horizontal coordinates correspond with the predictors. Parameter 0 is the intervention indicator, parameters 1-6 are height to age (HAZ) , weight to age (WAZ), weight to height (WHZ), cognition, language and motoric development, respectively, each at 24 months. These are followed by the gut microbiota parameters at 36, then at 24 months. The data included a total of 293 potential predictors. Vertical coordinates 0-9,10-19, 20-29 and 30-39 show best fitting models with 4, 3, 2 and 1 terms, respectively. Intensity of the pixels increases with size of the model coefficients. We established the 10 best models with 1-4 predictors. Accordingly, the best 4-term models were selected from more than 300 million models. The dark vertical band with horizontal coordinate 5 shows that, with a few exceptions, the best models with 2-4 parameters include the language development of the children at 24 months. There are further bands at horizontal coordinates 220, 231 and 277, respectively. The bands at coordinates 220 and 277 correspond with abundances of *Coprococcus* and an unclassified genus, respectively, both at 24 months. The bands alternate, showing that the abundances of the two genera are never in one and the same model. This indicates that these abundances are correlated in the present dataset so that they offer alternative explanations to the data at hand. Band 231 corresponds to the abundance of *Clostridium* cluster XI at 24 months. No alternatives replacing this genus in the model are apparent. Finally, the figure shows predictors that enter the fitted models only occasionally. Thus, there is no powerful evidence that these predictors should be included in this regression model.

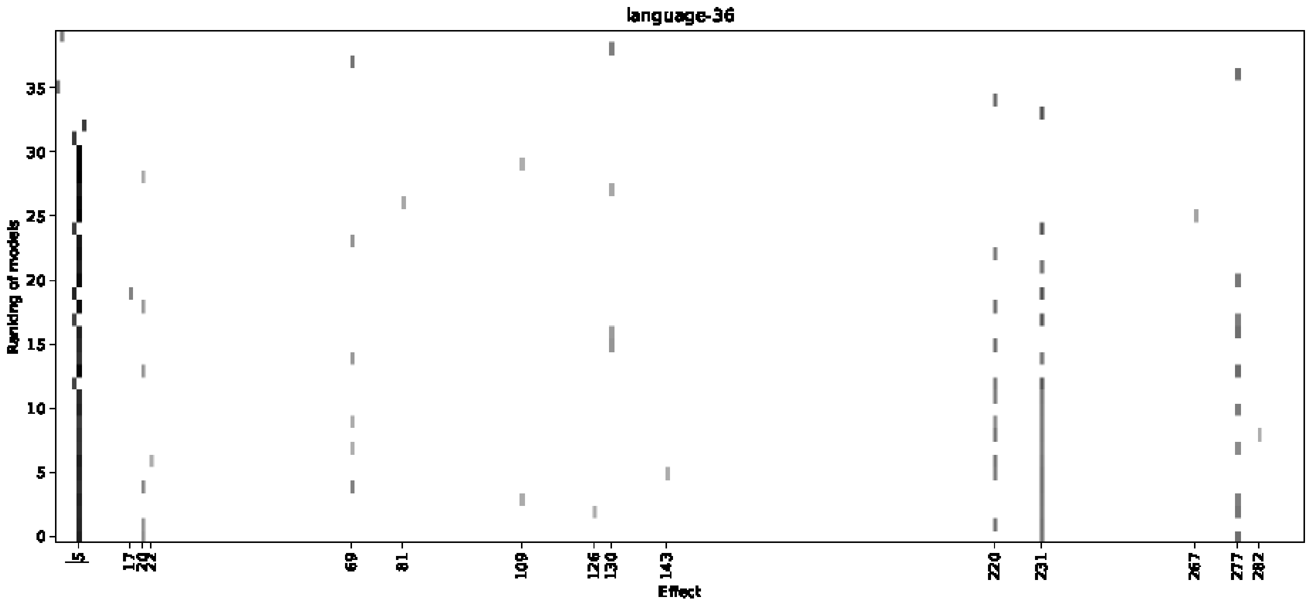

Supplement: Supplementary file 2 [file Data_Sheet_1.PDF]
